# Supplementary material for: The bromodomain-containing protein Ibd1 links multiple chromatin-related protein complexes to highly expressed genes in Tetrahymena thermophila
Source: Epigenetics Chromatin. 2018 Mar 9;11:10. doi: 10.1186/s13072-018-0180-6 (PMC5844071; doi:10.1186/s13072-018-0180-6)
Supplement: Supplementary file 1 — Additional file 1. Snf5_Alignments_Cloning. [file 13072_2018_180_MOESM1_ESM.pptx]

## Slide 1
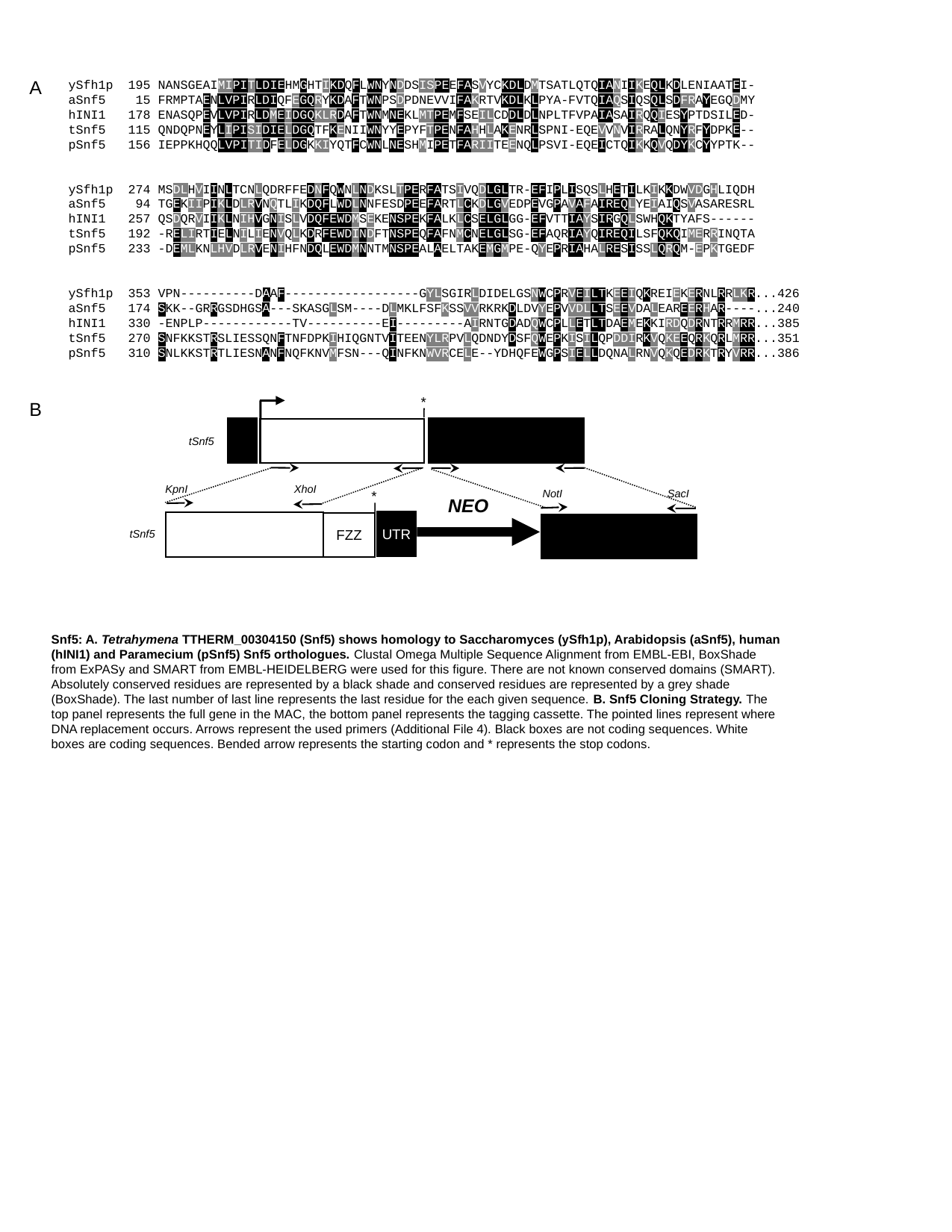

A
ySfh1p 195 NANSGEAIMIPITLDIEHMGHTIKDQFLWNYNDDSISPEEFASVYCKDLDMTSATLQTQIANIIKEQLKDLENIAATEI-
aSnf5 15 FRMPTAENLVPIRLDIQFEGQRYKDAFTWNPSDPDNEVVIFAKRTVKDLKLPYA-FVTQIAQSIQSQLSDFRAYEGQDMY
hINI1 178 ENASQPEVLVPIRLDMEIDGQKLRDAFTWNMNEKLMTPEMFSEILCDDLDLNPLTFVPAIASAIRQQIESYPTDSILED-
tSnf5 115 QNDQPNEYLIPISIDIELDGQTFKENIIWNYYEPYFTPENFAHHLAKENRLSPNI-EQEVVNVIRRALQNYRFYDPKE--
pSnf5 156 IEPPKHQQLVPITIDFELDGKKIYQTFCWNLNESHMIPETFARIITEENQLPSVI-EQEICTQIKKQVQDYKCYYPTK--
ySfh1p 274 MSDLHVIINLTCNLQDRFFEDNFQWNLNDKSLTPERFATSIVQDLGLTR-EFIPLISQSLHETILKIKKDWVDGHLIQDH
aSnf5 94 TGEKIIPIKLDLRVNQTLIKDQFLWDLNNFESDPEEFARTLCKDLGVEDPEVGPAVAFAIREQLYEIAIQSVASARESRL
hINI1 257 QSDQRVIIKLNIHVGNISLVDQFEWDMSEKENSPEKFALKLCSELGLGG-EFVTTIAYSIRGQLSWHQKTYAFS------
tSnf5 192 -RELIRTIELNILIENVQLKDRFEWDINDFTNSPEQFAFNMCNELGLSG-EFAQRIAYQIREQILSFQKQIMERRINQTA
pSnf5 233 -DEMLKNLHVDLRVENIHFNDQLEWDMNNTMNSPEALAELTAKEMGMPE-QYEPRIAHALRESISSLQRQM-EPKTGEDF
ySfh1p 353 VPN----------DAAF------------------GYLSGIRLDIDELGSNWCPRVEILTKEEIQKREIEKERNLRRLKR...426
aSnf5 174 SKK--GRRGSDHGSA---SKASGLSM----DLMKLFSFKSSVVRKRKDLDVYEPVVDLLTSEEVDALEAREERHAR----...240
hINI1 330 -ENPLP------------TV----------EI---------AIRNTGDADQWCPLLETLTDAEMEKKIRDQDRNTRRMRR...385
tSnf5 270 SNFKKSTRSLIESSQNFTNFDPKIHIQGNTVITEENYLRPVLQDNDYDSFQWEPKISILQPDDIRKVQKEEQRKQRLMRR...351
pSnf5 310 SNLKKSTRTLIESNANFNQFKNVMFSN---QINFKNWVRCELE--YDHQFEWGPSIELLDQNALRNVQKQEDRKTRYVRR...386
*
B
tSnf5
XhoI
KpnI
NotI
SacI
*
NEO
UTR
FZZ
tSnf5
Snf5: A. Tetrahymena TTHERM_00304150 (Snf5) shows homology to Saccharomyces (ySfh1p), Arabidopsis (aSnf5), human (hINI1) and Paramecium (pSnf5) Snf5 orthologues. Clustal Omega Multiple Sequence Alignment from EMBL-EBI, BoxShade from ExPASy and SMART from EMBL-HEIDELBERG were used for this figure. There are not known conserved domains (SMART). Absolutely conserved residues are represented by a black shade and conserved residues are represented by a grey shade (BoxShade). The last number of last line represents the last residue for the each given sequence. B. Snf5 Cloning Strategy. The top panel represents the full gene in the MAC, the bottom panel represents the tagging cassette. The pointed lines represent where DNA replacement occurs. Arrows represent the used primers (Additional File 4). Black boxes are not coding sequences. White boxes are coding sequences. Bended arrow represents the starting codon and * represents the stop codons.
