# Supplementary material for: The bromodomain-containing protein Ibd1 links multiple chromatin-related protein complexes to highly expressed genes in Tetrahymena thermophila
Source: Epigenetics Chromatin. 2018 Mar 9;11:10. doi: 10.1186/s13072-018-0180-6 (PMC5844071; doi:10.1186/s13072-018-0180-6)
Supplement: Supplementary file 2 — Additional file 2. Saf5_Alignments_Cloning. [file 13072_2018_180_MOESM2_ESM.pptx]

## Slide 1
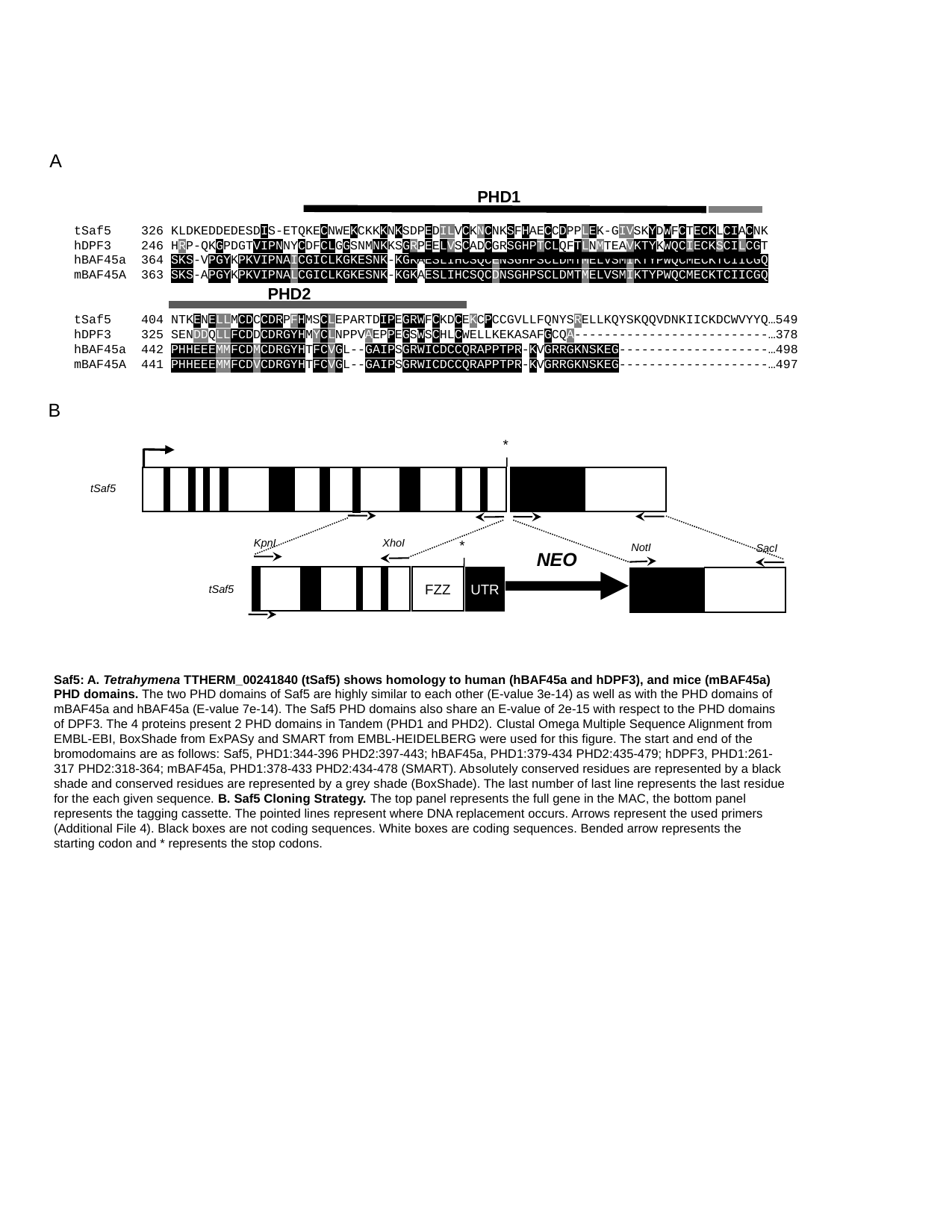

A
PHD1
tSaf5 326 KLDKEDDEDESDIS-ETQKECNWEKCKKKNKSDPEDILVCKNCNKSFHAECCDPPLEK-GIVSKYDWFCTECKLCIACNK
hDPF3 246 HRP-QKGPDGTVIPNNYCDFCLGGSNMNKKSGRPEELVSCADCGRSGHPTCLQFTLNMTEAVKTYKWQCIECKSCILCGT
hBAF45a 364 SKS-VPGYKPKVIPNAICGICLKGKESNK-KGKAESLIHCSQCENSGHPSCLDMTMELVSMIKTYPWQCMECKTCIICGQ
mBAF45A 363 SKS-APGYKPKVIPNALCGICLKGKESNK-KGKAESLIHCSQCDNSGHPSCLDMTMELVSMIKTYPWQCMECKTCIICGQ
tSaf5 404 NTKENELLMCDCCDRPFHMSCLEPARTDIPEGRWFCKDCEKCPCCGVLLFQNYSRELLKQYSKQQVDNKIICKDCWVYYQ…549
hDPF3 325 SENDDQLLFCDDCDRGYHMYCLNPPVAEPPEGSWSCHLCWELLKEKASAFGCQA--------------------------…378
hBAF45a 442 PHHEEEMMFCDMCDRGYHTFCVGL--GAIPSGRWICDCCQRAPPTPR-KVGRRGKNSKEG--------------------…498
mBAF45A 441 PHHEEEMMFCDVCDRGYHTFCVGL--GAIPSGRWICDCCQRAPPTPR-KVGRRGKNSKEG--------------------…497
PHD2
B
*
tSaf5
XhoI
KpnI
*
NotI
SacI
NEO
FZZ
UTR
tSaf5
Saf5: A. Tetrahymena TTHERM_00241840 (tSaf5) shows homology to human (hBAF45a and hDPF3), and mice (mBAF45a) PHD domains. The two PHD domains of Saf5 are highly similar to each other (E-value 3e-14) as well as with the PHD domains of mBAF45a and hBAF45a (E-value 7e-14). The Saf5 PHD domains also share an E-value of 2e-15 with respect to the PHD domains of DPF3. The 4 proteins present 2 PHD domains in Tandem (PHD1 and PHD2). Clustal Omega Multiple Sequence Alignment from EMBL-EBI, BoxShade from ExPASy and SMART from EMBL-HEIDELBERG were used for this figure. The start and end of the bromodomains are as follows: Saf5, PHD1:344-396 PHD2:397-443; hBAF45a, PHD1:379-434 PHD2:435-479; hDPF3, PHD1:261-317 PHD2:318-364; mBAF45a, PHD1:378-433 PHD2:434-478 (SMART). Absolutely conserved residues are represented by a black shade and conserved residues are represented by a grey shade (BoxShade). The last number of last line represents the last residue for the each given sequence. B. Saf5 Cloning Strategy. The top panel represents the full gene in the MAC, the bottom panel represents the tagging cassette. The pointed lines represent where DNA replacement occurs. Arrows represent the used primers (Additional File 4). Black boxes are not coding sequences. White boxes are coding sequences. Bended arrow represents the starting codon and * represents the stop codons.
