# Supplementary material for: The bromodomain-containing protein Ibd1 links multiple chromatin-related protein complexes to highly expressed genes in Tetrahymena thermophila
Source: Epigenetics Chromatin. 2018 Mar 9;11:10. doi: 10.1186/s13072-018-0180-6 (PMC5844071; doi:10.1186/s13072-018-0180-6)
Supplement: Supplementary file 3 — Additional file 3. Ibd1_Alignments_Cloning. [file 13072_2018_180_MOESM3_ESM.pptx]

## Slide 1
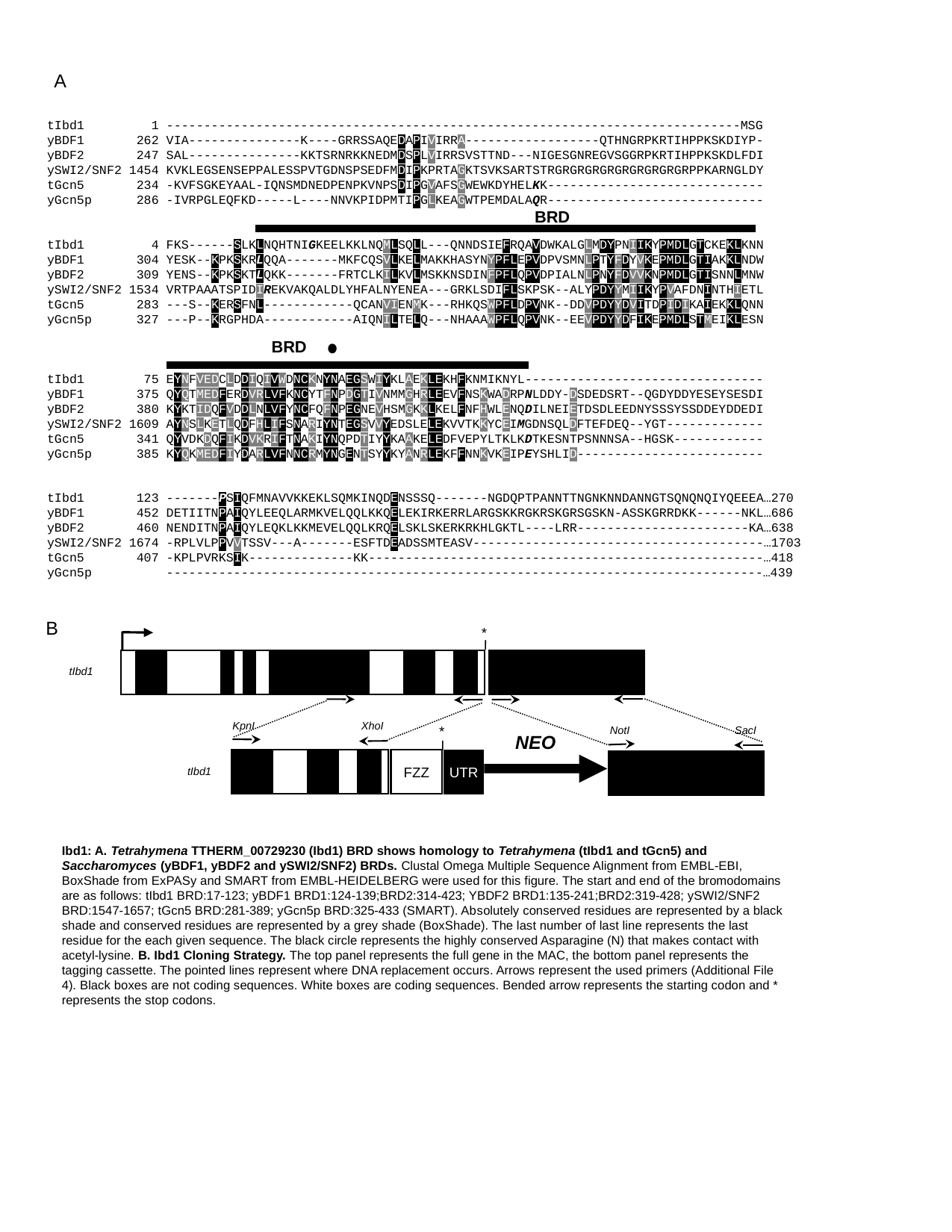

A
tIbd1 1 -----------------------------------------------------------------------------MSG
yBDF1 262 VIA---------------K----GRRSSAQEDAPIVIRRA------------------QTHNGRPKRTIHPPKSKDIYP-
yBDF2 247 SAL---------------KKTSRNRKKNEDMDSPLVIRRSVSTTND---NIGESGNREGVSGGRPKRTIHPPKSKDLFDI
ySWI2/SNF2 1454 KVKLEGSENSEPPALESSPVTGDNSPSEDFMDIPKPRTAGKTSVKSARTSTRGRGRGRGRGRGRGRGRGRPPKARNGLDY
tGcn5 234 -KVFSGKEYAAL-IQNSMDNEDPENPKVNPSDIPGVAFSGWEWKDYHELKK-----------------------------
yGcn5p 286 -IVRPGLEQFKD-----L----NNVKPIDPMTIPGLKEAGWTPEMDALAQR-----------------------------
tIbd1 4 FKS------SLKLNQHTNIGKEELKKLNQMLSQLL---QNNDSIEFRQAVDWKALGLMDYPNIIKYPMDLGTCKEKLKNN
yBDF1 304 YESK--KPKSKRLQQA-------MKFCQSVLKELMAKKHASYNYPFLEPVDPVSMNLPTYFDYVKEPMDLGTIAKKLNDW
yBDF2 309 YENS--KPKSKTLQKK-------FRTCLKILKVLMSKKNSDINFPFLQPVDPIALNLPNYFDVVKNPMDLGTISNNLMNW
ySWI2/SNF2 1534 VRTPAAATSPIDIREKVAKQALDLYHFALNYENEA---GRKLSDIFLSKPSK--ALYPDYYMIIKYPVAFDNINTHIETL
tGcn5 283 ---S--KERSFNL------------QCANVIENMK---RHKQSWPFLDPVNK--DDVPDYYDVITDPIDIKAIEKKLQNN
yGcn5p 327 ---P--KRGPHDA------------AIQNILTELQ---NHAAAWPFLQPVNK--EEVPDYYDFIKEPMDLSTMEIKLESN
tIbd1 75 EYNFVEDCLDDIQIVWDNCKNYNAEGSWIYKLAEKLEKHFKNMIKNYL--------------------------------
yBDF1 375 QYQTMEDFERDVRLVFKNCYTFNPDGTIVNMMGHRLEEVFNSKWADRPNLDDY-DSDEDSRT--QGDYDDYESEYSESDI
yBDF2 380 KYKTIDQFVDDLNLVFYNCFQFNPEGNEVHSMGKKLKELFNFHWLENQDILNEIETDSDLEEDNYSSSYSSDDEYDDEDI
ySWI2/SNF2 1609 AYNSLKETLQDFHLIFSNARIYNTEGSVVYEDSLELEKVVTKKYCEIMGDNSQLDFTEFDEQ--YGT-------------
tGcn5 341 QYVDKDQFIKDVKRIFTNAKIYNQPDTIYYKAAKELEDFVEPYLTKLKDTKESNTPSNNNSA--HGSK------------
yGcn5p 385 KYQKMEDFIYDARLVFNNCRMYNGENTSYYKYANRLEKFFNNKVKEIPEYSHLID-------------------------
tIbd1 123 -------PSIQFMNAVVKKEKLSQMKINQDENSSSQ-------NGDQPTPANNTTNGNKNNDANNGTSQNQNQIYQEEEA…270
yBDF1 452 DETIITNPAIQYLEEQLARMKVELQQLKKQELEKIRKERRLARGSKKRGKRSKGRSGSKN-ASSKGRRDKK------NKL…686
yBDF2 460 NENDITNPAIQYLEQKLKKMEVELQQLKRQELSKLSKERKRKHLGKTL----LRR-----------------------KA…638
ySWI2/SNF2 1674 -RPLVLPPVVTSSV---A-------ESFTDEADSSMTEASV---------------------------------------…1703
tGcn5 407 -KPLPVRKSIK--------------KK-----------------------------------------------------…418
yGcn5p --------------------------------------------------------------------------------…439
BRD
BRD
B
*
tIbd1
XhoI
KpnI
*
NotI
SacI
NEO
FZZ
UTR
tIbd1
Ibd1: A. Tetrahymena TTHERM_00729230 (Ibd1) BRD shows homology to Tetrahymena (tIbd1 and tGcn5) and Saccharomyces (yBDF1, yBDF2 and ySWI2/SNF2) BRDs. Clustal Omega Multiple Sequence Alignment from EMBL-EBI, BoxShade from ExPASy and SMART from EMBL-HEIDELBERG were used for this figure. The start and end of the bromodomains are as follows: tIbd1 BRD:17-123; yBDF1 BRD1:124-139;BRD2:314-423; YBDF2 BRD1:135-241;BRD2:319-428; ySWI2/SNF2 BRD:1547-1657; tGcn5 BRD:281-389; yGcn5p BRD:325-433 (SMART). Absolutely conserved residues are represented by a black shade and conserved residues are represented by a grey shade (BoxShade). The last number of last line represents the last residue for the each given sequence. The black circle represents the highly conserved Asparagine (N) that makes contact with acetyl-lysine. B. Ibd1 Cloning Strategy. The top panel represents the full gene in the MAC, the bottom panel represents the tagging cassette. The pointed lines represent where DNA replacement occurs. Arrows represent the used primers (Additional File 4). Black boxes are not coding sequences. White boxes are coding sequences. Bended arrow represents the starting codon and * represents the stop codons.
