# Supplementary material for: The bromodomain-containing protein Ibd1 links multiple chromatin-related protein complexes to highly expressed genes in Tetrahymena thermophila
Source: Epigenetics Chromatin. 2018 Mar 9;11:10. doi: 10.1186/s13072-018-0180-6 (PMC5844071; doi:10.1186/s13072-018-0180-6)
Supplement: Supplementary file 10 — Additional file 10. Additional_Methods. [file 13072_2018_180_MOESM10_ESM.docx]

**ADDITIONAL METHODS**

**Generation of WCE and Western blotting**

Whole-cell extracts (WCE) were prepared using 10% trichloroacetic acid in ice for 30 minutes. [1]. The WCEs were re-suspended in 90μL of SDS loading dye. To neutralize the solution, 10μL of 1M Tris was added. WCEs were separated by electrophoresis through 10% SDS-PAGE, transferred to nitrocellulose, and blotted with the indicated antibodies. Antibodies and dilutions used were anti-Flag (1:4000; Sigma), anti-Pdd1 (1:2500; Abcam), anti-Brg1 (1:1000, [2])

**Affinity purification and sample preparation**

Frozen cell pellets from ~500 ml of vegetatively growing *T. thermophila* harvested at a density of 3 × 10^5^ cells/ml were used. The pellets were thawed and re-suspended in 10 mM Tris–HCl (pH 7.5), 1 mM MgCl_2_, 300 mM NaCl and 0.2% NP40 plus yeast protease inhibitors (Sigma). 500 units of benzonase nuclease (Sigma E8263) were added and extracts were incubated for 30 min at 4°C. WCEs were clarified by centrifugation at 16,000×g with soluble material incubated for 4 h in the presence of 50 μl of packed M2-agarose (Sigma) and incubated at 4°C for 3 h. The M2-agarose was washed once with 10 ml IPP300 (10 mM Tris–HCl pH 8.0, 300 mM NaCl, 0.1% NP40), two times with 5mL of IP100 buffer (10 mM Tris–HCl pH 8.0, 100 mM NaCl, 0.1% NP40), and two times with 5mL of IP100 buffer without detergent (10 mM Tris–HCl pH 8.0, 100 mM NaCl). Elution was performed with 500 ul of 0.5 M NH_4_OH rotating for 20 minutes at room temperature. Protein eluates were prepared for mass spectrometry acquisition as previously described [3]. Briefly, the eluates were dried using a speedvac and resuspended in 10 μL of 20 mM Tris-HCl pH8 before being digested with 0.75 μg of trypsin (Sigma) for ~ 15 hours at 37°C with mild agitation. An extra 0.25 μg of trypsin was added to each sample and they were incubated for an additional 3 h. Samples were acidified to a final concentration of 2% acetic acid and stored at -80°C until their acquisition on a mass spectrometer.

**Experimental design for mass spectrometry experiments**

For each analysis, at least two biological replicates of each bait were processed independently. These were analysed alongside negative controls in each batch of samples processed. *Tetrahymena* cells expressing no tagged bait (i.e. empty cells) were employed as control. To minimize carry-over issues, extensive washes were performed between each sample (see details for each instrumentation type); and the order of sample acquisition on the mass spectrometer was also reversed for the second biological replicate to avoid systematic bias. On the LTQ mass spectrometer, a freshly made column was used for each sample.

**Preparation of HPLC columns for mass spectrometry**

A spray tip was formed on a fused silica capillary column (0.75 μm ID, 350 μm OD) using a laser puller (program = 4; heat = 280, FIL = 0, VEL = 18, DEL = 200). 10 cm – 12 cm of C_18_ reversed-phase material (Reprosil-Pur 120 C_18_-AQ, 3 μm; Dr.Maisch HPLC GmbH, Germany) was packed in the column by pressure bomb in MeOH. The column was equilibrated in buffer A prior to sample loading.

**Mass spectrometry acquisition using LTQ-Orbitrap mass spectrometers**

5 μL of each sample was directly loaded at 400 nL/min onto the equilibrated HPLC column. The peptides were eluted from the column by a gradient generated by a NanoLC-Ultra 1D plus (Eksigent, Dublin CA) nano-pump and analyzed on a LTQ-Orbitrap classic or LTQ-Orbitrap Velos (Thermo Electron) equipped with a nanoelectrospray ion source (Proxeon, Thermo Scientific). The LTQ-Orbitrap classic or Velos instrument under Xcalibur 2.0 was operated in the data dependent mode to automatically switch between MS and up to 10 subsequent MS/MS acquisitions. Buffer A was 100% H_2_O, 0.1% formic acid; buffer B was 100% acetonitrile (Can), 0.1% formic acid. The HPLC gradient program delivered an ACN gradient over 125 minutes. For the first twenty minutes, the flow rate was 400 μL/min at 2% B. The flow rate was reduced to 200 μL/min and the fraction of solvent B increased in a linear fashion to 35% until 95.5 minutes. Solvent B was increased to 80% over 5 minutes and maintained at that level until 107 minutes. The mobile phase was reduced to 2% B until the end of the run (125 min). The parameters for Data Dependent Acquisition on the mass spectrometer were: 1 centroid MS (mass range 400–2000) followed by MS/MS on the 10 most abundant ions. General parameters were: activation type = CID, isolation width = 1 m/z, normalized collision energy = 35, activation Q = 0.25, activation time = 10 msec. For data dependent acquisition, the minimum threshold was 500, the repeat count = 1, repeat duration = 30 sec, exclusion size list = 500, exclusion duration = 30 sec, exclusion mass width (by mass) = low 0.03, high 0.03.

**Mass spectrometry acquisition using LTQ mass spectrometers:**

The re-suspended sample was bomb-loaded in its entirety on the equilibrated column. The column was washed off-line for 10 min in buffer A, then placed in-line with a LTQ mass spectrometer equipped with an Agilent 1100 pump with split flow, and either the Thermo source, or a Proxeon source. Buffer A is 2% ACN, 0.1% formic acid; buffer B is 98% ACN, 0.1% formic acid. The HPLC gradient program delivered an ACN gradient over 120 min (1-5% buffer B over 4 min, 5-40% buffer B over 100 min, 40-60% buffer B over 5 min, 60-100% buffer B over 5 min, hold buffer B at 100% 3 min, and 100-0% B in 2 min). The parameters for Data Dependent Acquisition on the mass spectrometer were: 1 centroid MS (mass range 400-2000) followed by MS/MS on the 5 most abundant ions. General parameters were: activation type = CID, isolation width = 3, normalized collision energy = 32, activation Q = 0.25, activation time = 30 msec, wideband activation. For data dependent acquisition, minimum threshold was 1000, the repeat count = 1, repeat duration = 30 sec, exclusion size list = 500, exclusion duration = 30 sec, exclusion mass width (by mass) = low 1.2, high 1.5.

**Data Dependent Acquisition MS analysis:**

Mass spectrometry data were stored, searched and analyzed using the ProHits laboratory information management system (LIMS) platform [4]. Within ProHits, Thermo Fisher scientific RAW mass spectrometry files were converted to mzML and mzXML using ProteoWizard (3.0.4468; [5]). The mzML and mzXML files were searched using Mascot (v2.3.02). The spectra were searched with the RefSeq database (version 45, January 24th, 2011) acquired from NCBI against a total of 24,770 *T. thermophila* sequences. For the files analyzed on the Orbitraps, charges +2, +3 and +4 were allowed and the parent mass tolerance was set at 12 ppm while the fragment bin tolerance was set at 0.6 amu. For files analyzed on the LTQ, the charges +1, +2 and +3 were considered, with the parent mass tolerance set at 3 amu and the fragments at 0.6 amu. Deamidated asparagine and glutamine and oxidized methionine were allowed as variable modifications. SAINTexpress version 3.61 [6] was used as a statistical tool to calculate the probability value of each potential protein-protein interaction from background contaminants using default parameters. For the LTQ vegetative samples, controls were compressed from 38 to 20 controls while the conjugating samples were compressed from 11 controls to 8, using a strategy first introduced in [7].

**MS Data Visualization**

Dot plots were generated using ProHits-viz (prohits-viz.lunenfeld.ca [8]). Interaction networks were generated using Cytoscape (V3.0.1; [9]) with edge weighted based on spectral counts and nodes manually organized.

**Cloning, expression and purification of *T. thermophila* synthetic Ibd1**

An optimized synthetic gene to express a full length Ibd1 protein in *Escherichia coli* BL21(DE3) was generated and provided in a pUC57 plasmid (BioBasics Inc.). The synthetic gene was amplified by PCR (An additional Excel file shows this in more detail, see Additional file 4) and cloned into pET28a in-frame to add an N-terminal 6x His-tag to Ibd1. This plasmid was used for transformation into *E. coli* BL21 (DE3). Transformants were grown in media containing kanamycin +/- IPTG. Whole cell extracts were made by sonicating cell pellets in SDS loading dye followed by heating in boiling water. The samples were separated by SDS-PAGE, and visualized by staining with Fast SeeBand protein staining solution (Gene Bio-Application). *E. coli* were grown overnight at 37°C, re-suspended in liquid culture and incubated until the OD_600_ reached approximately 0.6. The culture was induced with IPTG for 4 hours. Extraction of the 6xHis-Ibd1 was done using Ni-NTA Fast Start Kit (Qiagen). Each fraction was tested by adding SDS loading dye followed by heating in boiling water. The samples were separated by electrophoresis through SDS-PAGE, and visualized by staining with Fast SeeBand protein staining solution (Gene Bio-Application). The Qiagen buffer was exchanged by size exclusion chromatography (PD-10 Desalting Columns, GE Healthcare) with a buffer with a final concentration of 10% glycerol and 10mM Tris-HCl pH 7.4. To verify recovery, SDS-PAGE followed by staining of the gel by Fast SeeBand Protein stain solution (Gene Bio-Application) was performed.

**Histone Peptide Array**

A commercial human histone peptide array (MODified Histone Peptide Array, Active Motif Catalog number 13005) and its reagents (MODified Protein Domain Binding Kit, Active Motif Catalog number 13007) was used to screen Ibd1 for binding interactions with 384 unique posttranslational modification combinations. The array contained 384 sites, each with a different combination of PTM. The mechanism of the array was comparable to a Western blot. The array was blocked with 5% milk for 1 hour, washed with 1xTBS and incubated with 6xHis-Ibd1, for 1 hour. The array was washed with 1xTBS and incubated first with the primary antibody, anti-6xHis, and then with the secondary antibody; anti-rabbit HRP. After washing again with 1xTBS, ECL was used for detection. The array was visualized by an imaging system (Gel Doc XR System, BioRad). The image obtained was processed in the software provided with the kit (Array Analyze Software, Active Motif). The output of the software was used to analyze each of the sites with binding interactions.

**Indirect immunofluorescence**

The cells were harvested and fixed during vegetative growth, after 24 hours of starvation (10mM Tris-HCl pH7.4), and at 3, 4.5, 6 and 8 hours post starvation mixing for indirect immunofluorescence as described [3]. A total of 20μL of each sample was mounted on slides with coverslips and epi-fluorescence examined on a compound, upright Leica DM5000B microscope under magnification of 400x.

**Identification of *Tetrahymena* BRD-containing proteins**

The *Tetrahymena* genome database (www.ciliate.org, [10]) was searched using the amino acid sequence of human and yeast bromodomains. The amino acid sequences of the identified *Tetrahymena* BRD-containing proteins were analysed using the Pfam database (http://pfam.sanger.ac.uk/) and SMART web tool (http://smart.embl-heidelberg.de/) for the domain identification. Phylogenetic analysis was carried out using the amino acid sequences of the BRD-containing domains found within the identified proteins. Sequences were aligned using MUSCLE [11]. The neighbour joining method using p-distances was employed to construct the phylogenetic tree (1000 bootstrap replicates) as implemented in MEGAv5.2.1 [12]. Tertiary structure for the BRD of Ibd1 was predicted at I TASSER server (http://zhanglab.ccmb.med.umich.edu/I-TASSER/) [13]. The superimposition of the Ibd1 BRD predicted structure was also carried out at I TASSER server using the BRD of human SMARCA2 protein (PDB: 5DKC).

**Chromatin Immunoprecipitation (ChIP)**

90 mL of Ibd1-FZZ cells in exponential growth were collected and cross-linked with 2.5 mL of 37% formaldehyde for 30 min at room temperature and neutralized with 13mL of 2.5M glycine. The chromatin was washed with 10mM Tris pH7.4 followed with cold lysis buffer (50mM Tris pH 8.0, 5mM EDTA, 1% SDS in H2O plus protease inhibitor (cOmplete™ Protease Inhibitor Cocktail) and 100mM PMSF). The chromatin was fragmented to 400 to 500 base pairs on ice by sonication 10 cycles 25 sec settings duty cycle 30%, output 0.3 (Branson 450 Analog Sonifier). The lysis buffer containing the sonicated chromatin was diluted 10 times using dilution buffer (50mM Tris pH 8.0, 100mM NaCl, 5mM EDTA, 2% Triton X and 0.2% deoxycholate. Chromatin immunoprecipation was performed using 25uL of packed bead volume M2 agarose beads (Sigma) rotating at 4ᵒC for 4hrs. The beads were wash one time with each of the following buffers 1X FA, 1X FA plus 500mM NaCl, LiCl buffer and 1X TE. DNA elution started in 2X Proteinase K buffer (20mM Tris pH8, 10mM EDTA and 1% SDS in H_2_O) at 65ᵒC for 20 minutes and diluted to 1X Proteinase K by H2O addition. RNase (Fermentas) was added an incubated for 1hr at 37ᵒC. Proteinase K (Fermentas) was added and incubated at 42ᵒC for 2 hrs and 65ᵒC for 8hrs. The DNA was isolated (Qiagen PCR purification kit) and H_2_O was used as elution.

**Next Generation Sequencing**

We performed 2 biological replicate analyses of our INPUT and IP samples. Library preparation was​ performed using Illumina​'s​ ​TruSeq ChIP Sample Preparation Kit ​(cat# IP-202-1012). ​Samples were ​sequenced on​ ​the Illumina HiSeq2500 ​platform as follows: single-end 58bp read length using v4 chemistry. The samples were d​e​multiplexed​ using​ ​bcl2fastq2 Conversion Software v2.17 (https://support.illumina.com/sequencing/sequencing_software/bcl2fastq-conversion-software.html) allowing for 1 barcode mismatch​. ​Passed Filter reads were use​ in downstream sample analysis. NGS was performed by the Donnelly Sequencing Centre, CSPro at University of Toronto.

**NGS Data Processing**

For each step of the NGS data processing we followed the method described by (Ponce M, Saettone A, et al., in preparation 2017), we used the data from the 2 NGS biological repetitions, each of which had an INPUT and an Immunoprecipitated (IP) sample.

*High Performance Computing:* The computations for the NGS data were performed on the General-Purpose Cluster (GPC) at the SciNet HPC Consortium at the University of Toronto. The data were assessed for quality using fastQC version 0.11.5 (Babraham Bioinformatics). After assessing for quality, we used Burrows-Wheeler Alignment (BWA) module 0.7.13 [14] and Sequence Alignment/Map (SAMtools) module 1.3.1 [15] to align and sort the raw data obtained from NGS, with the gene annotations and the *Tetrahymena* MAC genome, www.ciliate.org, [10].

*Peaks calling:* To call the peaks from the genic (ORF) and intergenic regions we respectively counted and extracted the Percentage of Clusters Passing Filters (PF clusters) that aligned to all ~25,000 annotated *T. thermophila* ORF and intergenic regions from the INPUT and IP. This was carried out using a “for”-loop combining tools from SAMtools (eg. “samtools view”) and linux shell commands (eg. “wc” for counting hits as lines) and deposited in the respective READS columns of a spreadsheet deposited in GSE103318. We also called peaks using MACS2 and we found that results are comparable, these peaks were deposited in GSE103318. Different to MACS2 that calls all peaks regardless of their position, genic or intergenic, (Ponce M, Saettone A, et al., in preparation 2017) pipeline calls peaks from either genic or intergenic regions and thus this pipeline is more suitable to answer our biological questions regarding position of Ibd1 in the genome.

*Peaks normalization:* The number of extracted reads per each given ORF or intergenic region represents a peak. These data were normalized 2 times. Since each of the 4 samples (2 sets of one INPUT and its respective IPs) from NGS has different amount of PF clusters, we first normalized these reads with respect to the smallest PF cluster from the Flowcell summary tab and deposited them in the RAW PEAKS columns of the same spreadsheet (GSE103318). In addition, since the size of each annotated gene and intergenic region are different, we further normalized the raw peaks based on their genic and intergenic region sizes with respect to the smallest gene (150b) and intergenic (12bp) region respectively and deposited them in the PEAKS columns of the same spreadsheet (GSE103318).

*Fold Enrichment:* After the 2 sequential normalizations of the INPUT and IP peaks, we calculated the fold enrichment for genic and intergenic regions of the 2 sample sets as the IP PEAK divided by the INPUT PEAK (IP/INPUT) and deposited them in the Fold enrichment columns of the same spreadsheet deposited in GSE103318. The average of both fold enrichment columns was calculated and deposited in the Average Fold Enrichment column of the same spreadsheet and ranked them.

**Analyses of Processed NGS Data**

*Table generation*: We obtained all data points, with fold enrichment that were greater than or equal to 2 in the Average Fold Enrichment column, from the data deposited in GSE103318 and placed the data into two Excel files. The first file contains 1607 genic regions and the second 2640 intergenic regions. This data was deposited in the additional Excel file 7 and 8, All_>2X_Fold_Enrichment tabs respectively.

*Strong vs Weak Peaks (Peaks cut-off)*: Since our interest was only to obtain strong peaks, we determined the weak peaks for further elimination. We generated the averaged peaks column for each data set and deposited in the additional Excel file 7 and 8, All_>2X_Fold_Enrichment tabs respectively. Very strong peak outliers were manually identified for the ORF and intergenic regions since they were presented in clear large amounts (i.e. >10X) with respect to the next following strongest peaks. The filtered outliers from the ORF table were TTHERM_02141639, TTHERM_02641280, TTHERM_02653301. From the intergenic regions table, we filtered the corresponding upstream and downstream intergenic regions of the filtered ORF. We did not use these genic and intergenic regions for the cut-off calculation, however we used them for following calculations. We next transferred this data to a new tab to calculate the values distribution (An additional Excel file shows this in more detail, see Additional file 7 and 8, Avg_Peak_Values_Distribution tabs). With these data, we generated a bell curve and two bin ranges, one with random numbers generated by Excel and the other with the original data. We designated peak values corresponding to the lower 5% limit with respect to the highest averaged peak value as background or weak peaks. The weak peaks were filtered for all further calculations. We showed the cut-off values on the Bin (Average IP_N2 Peak) curves in red (An additional Excel file shows this in more detail, see Additional file 7 and 8, Avg_Peak_Values_Distribution tabs). The calculations were carried out as follows: i] for the ORF, the lower limit with respect to the highest peak value of 4957 reads was calculated as 4957x5/100=247.85. As a result, all ORF that have an average IP_N2 peak lower than 247.85 were filtered; ii] for the intergenic regions, the lower limit with respect to highest peak value of 222 reads was calculated as 222x5/100=11.1. As a result, all intergenic regions that have an average IP_N2 peak lower than 11.1 were filtered. The obtained data for the genic and intergenic regions were deposited in a new tab called >2X_Enriched_with_Strong_Peaks in the Additional file 7 and 8. In addition, the corresponding TTHERM value obtained from GSM692081[16] for the genic region were deposited in the same tab. The GSM692081 data was obtained after RNA-Seq analyses and it shows the gene expression of the ~25,000 predicted genes encoded by the *Tetrahymena* MAC during vegetative growth [16]. In [16], it was also concluded that the RNA-Seq and microarray data [17] present a strong correlation. It addition, the RNA-Seq analysis uncovered more genes that were thought to be unexpressed by the microarray data. Thus, bringing this number from 22% (microarray data) to 3.8% (RNA-Seq data). From the RNA-Seq data [16] and for our analyses, we stablished that genes that have more than 1000 and between 1000 and 100 RNA mapped reads during vegetative growth are considered highly and moderately expressed respectively. This data was deposited in the additional Excel file 7, >2X_Enriched_with_Strong_Peaks and RNA-Seq tabs.

*Reproducibility:* The biological replicates confidence value was calculated for the data presented in the additional Excel file 7 and 8, >2X_Enriched_with_Strong_Peaks tabs at 95% confidence and deposited in the additional Excel file 7 and 8, Replicates_reproducibility tabs respectively. Based on the data distribution we noticed that the experiment reproducibility, between the two repetitions, is between 61% to 99.5% for the ORF and 49% to 99.7% for the intergenic regions. We assumed that this variability is expected due to the differences in cell physiology or ChIP-Seq related variations. Pearson correlation showed 0.91 for ORF and 0.71 for intergenic regions showing a strong positive association between both replicates.

*Peaks validation through visual inspection:* IGV [18] was used to visually inspect and validate the obtained ranked fold enrichment data points from the Additional file 7 and 8, >2X_Enriched_with_Strong_Peaks tabs.

*Localization of Ibd1 to genic regions and highly expressed genes:* To determine Ibd1 localization based on its localization at highly expressed genes, we grouped the data obtained from the additional Excel file 7, >2X_Enriched_with_Strong_Peaks tab, based on their enrichment (≥ 2X, ≥ 4X, ≥ 6X and ≥ 8X) and their respective highly expressed, low expressed state and non-available TTHERMs for the GSM692081 data set. This analysis was deposited in the additional Excel file 7, localization tab.

*Ibd1 occupancy*: To determine the preference of Ibd1 for genic or intergenic regions we grouped the data obtained from the additional Excel file 8, >2X_Enriched_with_Strong_Peaks tab based on their enrichment (≥ 2X, ≥ 4X, ≥ 6X and ≥ 8X) and compared to the data obtained from the additional Excel file 7, localization tab. *Assigning putative function to peaks populations with values ≥4X* based on their genomic position*:* Since peaks that are ≥4-fold enriched in the ORF and intergenic regions are clearly enriched, we filtered the values from the additional Excel file 7 localization tab and the additional Excel file 8, Ibd1_localization tab that are below 4. The data obtained for the genic and intergenic regions that had values ≥4X were deposited in the additional Excel file 7 and 8, 4X_+_Ibd1_Occupancy tabs. For the ORF (>2X_Enriched_with_Strong_Peaks and 4X_+_Ibd1_Occupancy tabs from Additional_file_7_ChIP_seq_ORF) and *Tetrahymena* genes data (as presented in GSE103318_BD1_BD2_ORF_peaks.xlsx file from GEO accession GSE103318) we used STRING (https://string-db.org/) [19] to predicted their biological function based on the Gene Ontology (Biological Process) [20]. The data was uploaded to STRING in batches of 2000 TTHERMS (or less) each time. This data was deposited in the Additional file 7, 2X_+_GO_Biological_Process, 4X_+_GO_Biological_Expression and AllTtGenes_GO_Biological_Proces tabs respectively. For the intergenic region we used IGV [18] to visually inspect and localize neighbouring TTHERM numbers to determine Ibd1 putative function based on the accumulation of each peak throughout the genome with respect to promoters and/or terminators an deposited them in An additional Excel file 8, Intergenic_Groups tab. Thus, if the Ibd1 enrichment was neighboring a 5’ region we group these as promoter and if it was closer to a 3’ ORF region, we grouped them as terminators. We used IGV [18] to visually inspect and localize neighbouring TTHERM numbers and deposited these in the same tab. We next extracted de data from the additional Excel file 8, Intergenic_Groups tab, that contains ≥4X values for ORF and intergenic regions and deposited this data in the additional Excel file 8, Combining_Intergenic_and_ORF tab.

**REFERENCES**

1. Bright LJ, Kambesis N, Nelson SB, Jeong B, Turkewitz AP. Comprehensive analysis reveals dynamic and evolutionary plasticity of Rab GTPases and membrane traffic in Tetrahymena thermophila. PLoS Genet. 2010;6:1–18.

2. Fillingham J, Garg J, Tsao N, Vythilingum N, Nishikawa T, Pearlman RE. Molecular genetic analysis of an SNF2/brahma-related gene in Tetrahymena thermophila suggests roles in growth and nuclear development. Eukaryot. Cell. 2006;5:1347–59.

3. Garg J, Lambert J-P, Karsou A, Marquez S, Nabeel-Shah S, Bertucci V, et al. Conserved Asf1-importin β physical interaction in growth and sexual development in the ciliate Tetrahymena thermophila. J. Proteomics. Elsevier B.V.; 2013;94C:311–26.

4. Liu G, Knight JDR, Zhang JP, Tsou CC, Wang J, Lambert JP, et al. Data Independent Acquisition analysis in ProHits 4.0. J. Proteomics. Elsevier B.V.; 2016;149:64–8.

5. Kessner D, Chambers M, Burke R, Agus D, Mallick P. ProteoWizard: Open source software for rapid proteomics tools development. Bioinformatics. 2008;24:2534–6.

6. Teo G, Liu G, Zhang J, Nesvizhskii AI, Gingras AC, Choi H. SAINTexpress: Improvements and additional features in Significance Analysis of INTeractome software. J. Proteomics. Elsevier B.V.; 2014;100:37–43.

7. Mellacheruvu D, Wright Z, Couzens AL, Lambert J, St-denis N, Li T, et al. The CRAPome: a Contaminant Repository for Affinity Purification Mass Spectrometry Data. Nat Methods. 2013;10:730–6.

8. Knight, J.D., Liu G, Zhang JP, Pasculescu A, Choi H, Gingras AC. A web-tool for visualizing quantitative protein-protein interaction data. Proteomics. 2015;15:1432–6.

9. Shannon P, Markiel A, Owen Ozier 2, Baliga NS, Wang JT, Ramage D, et al. Cytoscape: a software environment for integrated models of biomolecular interaction networks. Genome Res. 2003;2498–504.

10. Stover NA, Krieger CJ, Binkley G, Dong Q, Fisk DG, Nash R, et al. Tetrahymena Genome Database (TGD): a new genomic resource for Tetrahymena thermophila research. Nucleic Acids Res. 2006;34:D500-3.

11. Edgar RC. MUSCLE: a multiple sequence alignment method with reduced time and space complexity. BMC Bioinformatics. 2004;5:113.

12. Tamura K, Peterson D, Peterson N, Stecher G, Nei M, Kumar S. MEGA5: Molecular evolutionary genetics analysis using maximum likelihood, evolutionary distance, and maximum parsimony methods. Mol. Biol. Evol. 2011;28:2731–9.

13. Roy A, Kucukural A, Zhang Y. I-TASSER: a unified platform for automated protein structure and function prediction. Nat Protoc. 2010;5:725–38.

14. Li H, Durbin R. Fast and accurate short read alignment with Burrows-Wheeler transform. Bioinformatics. 2009;25:1754–60.

15. Li H, Handsaker B, Wysoker A, Fennell T, Ruan J, Homer N, et al. The Sequence Alignment/Map format and SAMtools. Bioinformatics. 2009;25:2078–9.

16. Xiong J, Lu X, Zhou Z, Chang Y, Yuan D, Tian M, et al. Transcriptome analysis of the model protozoan, tetrahymena thermophila, using deep RNA sequencing. PLoS One. 2012;7:1–13.

17. Miao W, Xiong J, Bowen J, Wang W, Liu Y, Braguinets O, et al. Microarray Analyses of Gene Expression during the Tetrahymena thermophila Life Cycle. PLoS One. 2009;4:e4429.

18. Robinson JT, Thorvaldsdóttir H, Winckler W, Guttman M, Lander ES, Getz G, et al. Integrative Genomics Viewer. Nat. Biotechnol. 2011;29:24–26.

19. Snel B, Lehmann G, Bork P, Huynen MA. STRING: a web-server to retrieve and display the repeatedly occurring neighbourhood of a gene. Nucleic Acids Res. 2000;28:3442–4.

20. Gene Ontology C. Gene ontology: Tool for the identification of biology. Nat. Genet. 2000;25:25–9.
